# Genetic diversity of *Leptospira* strains circulating in humans and dogs in France in 2019-2021

**DOI:** 10.3389/fcimb.2023.1236866

**Published:** 2023-08-17

**Authors:** Marta Garcia-Lopez, Celine Lorioux, Anais Soares, Sabine Trombert-Paolantoni, Elena Harran, Florence Ayral, Mathieu Picardeau, Zouheira Djelouadji, Pascale Bourhy

**Affiliations:** ^1^ Biology of Spirochetes Unit, National Reference Center for Leptospirosis, Institut Pasteur, Paris, France; ^2^ USC 1223-RS2GP, Laboratory of Leptospira and Veterinary Analysis, VetAgro Sup, University of Lyon, Marcy l’Etoile, France; ^3^ Department of Infectiology, Eurofins Biomnis, Lyon, France; ^4^ Cerba Laboratory, Saint-Ouen L’Aumône, France

**Keywords:** leptospirosis, zoonotic disease, human, dog, France, lfb1 gene

## Abstract

Leptospirosis is a bacterial zoonotic disease. Humans and dogs are susceptible hosts, with similar clinical manifestations ranging from a febrile phase to multiple organ dysfunction. The incidence of leptospirosis in mainland France is relatively high, at about 1 case per 100,000 inhabitants, but our knowledge of the strains circulating in humans and dogs remains limited. We studied the polymorphism of the *lfb1* gene sequences in an exhaustive database, to facilitate the identification of *Leptospira* strains. We identified 46 species-groups (SG) encompassing the eight pathogenic species of *Leptospira*. We sequenced the *lfb1* gene amplification products from 170 biological samples collected from 2019 to 2021: 110 from humans and 60 from dogs. Epidemiological data, including vaccination status in dogs, were also collected. Three *Leptospira* species displaying considerable diversity were identified: *L. interrogans*, with eight *lfb1* species-groups (including five new *lfb1* species-groups) in humans and dogs; *L. kirschneri*, with two *lfb1* species-groups in humans and dogs; and *L. borgpetersenii*, with one *lfb1* species-group in humans only. The *lfb1* species-group *L. interrogans* SG1, corresponding to serovar Icterohaemorrhagiae or Copenhageni, was frequently retrieved from both humans and dogs (*n=*67/110; 60.9% and *n=*59/60; 98.3% respectively). A high proportion of the affected dogs developed the disease despite vaccination (*n=*30/60; 50%). Genotyping with the polymorphic *lfb1* gene is both robust and simple. This approach provided the first global picture of the *Leptospira* strains responsible for acute infections in mainland France, based on biological samples but without the need for culture. Identification of the *Leptospira* strains circulating and their changes over time will facilitate more precise epidemiological monitoring of susceptible and reservoir species. It should also facilitate the monitoring of environmental contamination, making it possible to implement preventive measures and to reduce the burden of this disease.

## 1 Introduction

With an estimated one million cases of severe leptospirosis in humans each year, resulting in 60,000 deaths, leptospirosis is considered one of the commonest zoonoses worldwide. It has been recognized as an emerging global public health problem, because its incidence is increasing in both developing and developed countries (World Health Organization, 2003; Costa et al., 2015; Pijnacker et al., 2016; Bourhy et al., 2017). Leptospirosis is essentially considered a tropical disease, as high temperatures and humid climates favour the survival of the causal bacterium (Evangelista and Coburn, 2010; Costa et al., 2015), and it predominantly affects impoverished populations. Nevertheless, it is also widely reported in temperate areas, including Europe (Goarant, 2016; Pijnacker et al., 2016). Leptospirosis also has a major impact on the health of wild and domestic mammals, and can cause major economic losses in the livestock sector (Noguera et al., 2022). Dogs are particularly susceptible to leptospirosis, and vaccines are available to protect them from disease (Azócar-Aedo et al., 2014; Ellis, 2015).

The genus *Leptospira* is currently subdivided into 68 genomic species, including saprophytic and pathogenic species. Infections in humans and animals are caused by only eight pathogenic species*: L. interrogans, L. kirschneri, L. noguchi, L. santarosai, L. mayottensis, L. borgpetersenii, L. alexanderi* and *L. weilli* (Vincent et al., 2019). Serological classification based on polymorphism of the lipopolysaccharide (LPS) O-antigen has made it possible to identify more than 300 serovars (sv) grouped into 26 serogroups (sg) (Nieves C et al., 2023).

Infection with *Leptospira* spp. occurs predominantly through contact between abraded skin or mucous membranes and water or soil contaminated with the urine of infected animals, such as rodents, which are the main reservoir of human leptospirosis (Ellis, 2015). The rat (*Rattus* spp.) serves as a host for the most widespread serovars worldwide, Icterohaemorrhagiae and Copenhageni (Boey et al., 2019). Other studies have demonstrated the existence of several other hosts, such as mice, voles, and hedgehogs, harboring other *Leptospira* serovars (Ayral et al., 2016; Izquierdo-Rodríguez et al., 2020; Jeske et al., 2021). Moreover, livestock can also be a source of environmental contamination (Zarantonelli et al., 2018; Sykes et al., 2022).

Human leptospirosis is not a notifiable disease in France. The French National Reference Center (FNRC) conducts passive surveillance by compiling biologically confirmed cases through a network of partners (hospitals and diagnostic laboratories). The incidence of leptospirosis in mainland France is one of the highest in Europe, with approximately 1 case per 100,000 inhabitants or more than 600 cases per year (Bourhy et al., 2017). This disease is associated with working in contact with animals, environment-related activities and recreational activities linked to water (Nardone et al., 2004; Guillois et al., 2018; Velardo et al., 2022).

Information about the *Leptospira* strains circulating in susceptible species is scarce in France and, indeed, in Europe generally. There are several reasons for this. The first one is the laborious nature of *Leptospira* culture methods, which require fresh biological samples and reference laboratories capable of identifying the strains concerned. Moreover, molecular diagnostic methods, such as Polymerase Chain Reactions (PCR), are gradually supplanting serological tests, which remain the only available epidemiological tool for serogroup identification in *Leptospira* spp. There is, therefore, a crucial need for a new *Leptospira* genotyping method suitable for direct use on biological samples that is sensitive, discriminant, simple to implement and inexpensive. Identification of the strains in circulation is essential for diagnosis (to ensure that the diagnostic methods used are capable of detecting these strains), epidemiology (to characterise the reservoirs), surveillance (to detect the occurrence of new genotypes) and prevention (to evaluate the efficacy of vaccines and for the development of new vaccines).

Epidemiological studies of circulating strains are difficult to implement because of the lack of clinical isolates. Pathogenic *Leptospira* strains are slow-growing bacteria that can be grown only on complex culture media. Current knowledge about the epidemiology of leptospirosis is based on serological results obtained with the reference microscopic agglutination test (MAT), which can be used to identify the infecting serogroup (Faine and Stallman, 1982; Picardeau, 2020). However, this technique has known inconsistencies and weaknesses (Kusum et al., 2005; Smythe et al., 2009). Molecular techniques for studying *Leptospira* epidemiology have been described, including a core-genome MLST scheme (cgMLST) for isolates (Guglielmini et al., 2019). In the absence of isolates, several alternative methods are available for genotyping, including multispacer sequence typing or MST (Zilber et al., 2014), variable number tandem repeat or VNTR methods (Salaün et al., 2006), and multilocus sequence typing or MLST (Ahmed et al., 2006). Various genes, including the 16S rRNA*, lipL32, secY, lfb1*, and *lic12008* genes can also be sequenced for the direct genotyping of strains present in biological samples without the need for isolation in culture (Merien et al., 2005; Marquez et al., 2017; Santos et al., 2018).

Lfb1 is a putative adhesin of the “fibronectin-binding protein” family (Merien et al., 2000) and *lfb1* gene sequences have been shown to be congruent with the genomic species classification of pathogenic *Leptospira* strains (Merien et al., 2005). Perez et al. showed that the *lfb1* locus displayed greater phylogenetic polymorphism than the 16S rRNA gene, making it possible to identify strains down to subspecies level (Perez and Goarant, 2010). This identification method has since been used in numerous epidemiological studies in humans and/or animals (Grégoire et al., 2020; Soupé-Gilbert et al., 2022).

The FNRC has an exhaustive database of sequences of strains from many regions of the world and from a wide range of hosts. We used this database to evaluate the *lfb1* marker for large-scale use for identification purposes. The objectives of our study were to validate the robustness of the *lfb1* method for detecting and genotyping *Leptospira* strains directly from DNA and to propose a new classification system. We then applied this method to the genetic characterization of strains circulating in humans and dogs with acute leptospirosis in mainland France from 2019 to 2021. This work is completed to describe the incidence and geographical distribution in humans, and also the vaccination status in dogs.

## 2 Materials and methods

### 2.1 *lfb1*-derived phylogeny “reference classification”

*lfb1* sequences were extracted from the genomes of pathogenic species from the *Leptospira* cgMLST database (https://bigsdb.pasteur.fr/leptospira/). This publicly available web-based database currently contains sequences from 834 pathogenic *Leptospira* strains from different hosts around the world. A clonal group (CG) is defined as a group of cgMLST allelic profiles differing from at least one other member of the group by no more than 40 allelic mismatches over the 545 genetic loci (Guglielmini et al., 2019).

A phylogenetic tree was generated with BioNumerics V7.6 (Applied-Maths, Saint-Martens-Latem, Belgium). The 46 *lfb1* sequence strains for the production of the phylogenetic tree are summarized in **Table 1**. The *lfb1* nucleotide sequences have been deposited in Genbank under accession number OR101259 - OR101474.

**Table 1:** List of *Leptospira* species groups (SGs) defined on the basis of *lfb1* sequence polymorphism.

Species/Group (ID) *n*=46	Serogroup	Serovar reference strains	Number of genomes *n=*834	Number of different CGs *n=*227	Number CGs of cgMLST in BIGSdb
*L.interrogans* SG1 (245)	Icterohaemorrhagiae	Icterohaemorrhagiae/Copenhageni	119	1	6
*L.interrogans* SG2 (16)	Pyrogenes Autumnalis	Pyrogenes Bim	32	9	9/10/11/30/39/76/77/81/400
*L.interrogans* SG3 (1058)	Djasiman	Djasiman	4	1	37
*L.interrogans* SG4 (1052)	Grippotyphosa Pyrogenes Sejröe Icterohaemorrhagiae	Grippotyphosa/Muelleri Pyrogenes Wolfii/Hardjo/Hardjobovis Lai/Naam	56	28	2/3/16/19/27/29/34/112/166/176/180/187/238/240/ 275/278/279/280/285/289/290/294/296/302/303/307/311/386
*L.interrogans* SG5 (843)	Australis Bataviae Grippotyphosa	Bratislava/Lora/Jalna/Muenchen/Fugis Bataviae Valbuzzi	57	14	4/12/38/40/69/106/108/175/272/281/297/298/306/345
*L.interrogans* SG6 (25)	Grippotyphosa	Linhai	1	1	17
*L.interrogans* SG7 (37)	Pyrogenes	Manilae	7	1	2
*L.interrogans* SG8 (1121)	Canicola	Kuwait	4	1	349
*L.interrogans* SG9 (1055)	Hebdomadis	Hebdomadis	1	1	331
*L.interrogans* SG10 (478)	Canicola Pomona Sejröe	Canicola Pomona/Kennewicki Medanensis	111	11	5/26/28/41/75/277/284/287/288/300/322
*L.interrogans* SG11 (802)	Grippotyphosa	Grippotyphosa	2	2	31/295
*L.interrogans* SG12 (511)	Grippotyphosa	Unknown	1	1	228
*L.interrogans* SG13 (686)	Autumnalis Louisiana	Autumnalis Lanka	5	5	74/266/269/271/321
*L.kirschneri* SG1 (1046)	Grippotyphosa Cynopteri Pomona Mini Autumnalis Icterohaemorrhagiae	Grippotyphosa/Valbuzzi/Vanderhoedeni Unknown Pomona/Mozdok Unknown Bim Mwogolo/Sokoine	62	17	20/49/59/62/63/64/65/70/73/83/84/85/123/184/353/354/355
*L.kirschneri* SG2 (78)	Autumnalis Australis	Bulgarica Ramisi	6	6	42/43/57/178/299/323
*L.kirschneri* SG3 (1038)	Grippotyphosa	Grippotyphosa	1	1	305
*L.kirschneri* SG4 (304)	Pomona	Tsaratsovo	2	1	139
*L.kirschneri* SG5 (619)	Canicola Grippotyphosa	Unknown Ratnapura	10	3	22/129/185
*L.noguchi* SG1 (1048)	Louisiana Panama Australis	Louisiana Mangus Rushan	9	9	113/162/224/225/226/227/385/423/424
*L.noguchi* SG2 (254)	Australis	Unknown	3	3	114/115/116
*L.noguchi* SG3 (846)	Panama Australis Autumnalis	Panama Peruviana Autumnalis	5	5	55/99/125/168/193
*L.noguchi* SG4 (217)	Unknown	Unknown	1	1	100
*L.noguchi* SG5 (429)	Australis	Bajan/Barbudensis	4	1	179
*L.noguchi* SG6 (495)	Australis	Nicaragua	1	1	181
*L.noguchi* SG*7* (515)	Australis	Unknown	1	1	188
*L.borgpetersenii* SG1 (933)	Sejröe Ballum Javanica Mini	Sejröe Ballum/Castellonis/Kenya/Arborea/SarMini Javanica Mini/Szwajizak	193	12	8/15/24/6/78/165/267/309/313/318/348/352
*L.borgpetersenii* SG*2* (972)	Sejröe	Hardjo/Hardjobovis	11	1	72
*L.borgpetersenii* SG3 (205)	Sejröe	Unknown	9	1	98
*L.borgpeterseni*i SG4 (13)	Pyrogenes	Pyrogenes/Balcanica	4	4	7/312/356/426
*L.borgpetersenii* SG5 (152)	Pomona	Pomona	4	2	80/145
*L.borgpetersenii* SG6 (786)	Tarassovi	Tarassovi/Guidae	2	1	167
*L.alexanderi* SG1 (20)	Manhao	Manhao	1	1	13
*L.alexanderi* SG2 (1249)	Unknown	Unknown	1	1	402
*L. mayottensis* SG1 (178)	Unknown	Unknown	17	1	82
*L. mayottensis* SG2 (1239)	Unknown	Unknown	1	1	366
*L. mayottensis* SG3 (177)	Mini	Unknown	10	1	79
*L.weillii* SG1 (49)	Hebdomadis Javanica	Unknown Coxi	9	9	18/44/104/273/282/283/320/401/403
*L.weillii* SG2 (687)	Celledoni	Unknown	3	3	45/262/264
*L.weillii* SG3 (232)	Tarassovi Sarmin	Topaz Sarmin	6	6	46/47/109/163/308/346
*L.weillii* SG4 (79)	Unknown	Unknown	5	5	32/33/274/292/293
*L.santarosai* SG1 (1066)	Shermani Javanica Sejröe Grippotyphosa Hebdomadis	Shermani Arenal Sejröe/Guaricura Grippotyphosa Kambale	30	30	53/54/56/103/118/119/120/126/310/338/389/391/392/393/395/396/398/404/405/406/409/410/411/412/413/414/415/416/417/422/427
*L.santarosai* SG2 (1189)	Mini Pyrogenes Celledoni Tarassovi	Szwajizak Unknown Celledoni Corresdores	16	16	50/101/127/128/130/131/132/133/134/196/387/388/394/397/407/408
*L.santarosai* SG3 (90)	Grippotyphosa	Canalzonae	4	4	51/122/186/399
*L.santarosai* SG4 (91)	Javanica	Arenal	1	1	52
*L.santarosai* SG5 (220)	Unknown	Unknown	1	1	102
*L.santarosai* SG6 (1294)	Sarmin	Unknown	1	1	117

### 2.2 Samples from humans and dogs

PCR-positive samples collected from humans and dogs in mainland France between January 2019 and December 2021 were included in this study. Cases imported from French overseas territories were excluded from the study.

The samples studied were obtained from i) the routine diagnosis of human samples by real-time PCR (RT-PCR) SYBR-Green targeting *lfb1* (Merien et al., 2005), which is performed at the FNRC for Leptospirosis at the Pasteur Institute, and ii) dogs testing positive in routine diagnostic RT-PCR targeting the 16S rRNA *(rrs)* gene of pathogenic *Leptospira* strains (Waggoner et al., 2014) at the Laboratory of Leptospira and Veterinary Analysis (LAV) at VetAgro Sup (the Veterinary School of Lyon, France).

### 2.3 Data collection and ethics statement

The human samples and associated data were collected and used in the framework of the surveillance activities of the FNRC for Leptospirosis. These activities are performed in accordance with the mandate awarded to the FNRC by the French Ministry of Health and the French Public Health Code. Dog samples and associated data were collected and sent at by veterinarians from across the country in the context of leptospirosis suspicion. The confirmatory tests were performed by the LAV.

Associated clinical and epidemiological data are entered by clinicians on a document accompanying the samples. These data are often scarce, particularly for human infections. They included the sex and age of the human patient or dog, type of sample, sampling date, geographic information (zip code and region), and vaccination status for dogs. In the absence of geographic data for the patients/dogs, the address of the laboratory or the veterinary clinic was used assuming that the patients/dogs were exposed in the same region.

### 2.4 Human case definition/serogroup identification

A case was defined as an individual resident in mainland France at the time of infection with clinical findings suggestive of leptospirosis and either a Microscopic Agglutination Test (MAT) titer ≥100 for at least one pathogenic serovar or other laboratory results indicative of leptospirosis (ELISA for IgM, PCR or culture). The panel of strains used for MAT included the serovars Australis, Autumnalis, Bataviae, Canicola, Castellonis, Copenhageni, Cynopteri, Djasiman, Grippotyphosa, Hardjo, Hebdomadis, Icterohaemorrhagiae, Javanica, Louisiana, Mini, Panama, Pomona, Pyrogenes, Sarmi, Sejroe, Shermani and Tarassovi. The infecting serogroup was determined based on the serovar with the highest titer. If the highest titer was recorded for several different serovars, the serogroup was considered to be undetermined. The incidence of annual leptospirosis in humans was calculated with the French population data for 2019, 2020 and 2021 obtained from the National Institute of Statistics and Economic Studies (INSEE: https://www.insee.fr/).

The map of the estimated mean annual incidence of leptospirosis in humans by region and the distribution of the different infecting species-groups were performed with R Studio version 2022.12.0 software, produced with the GeoJSON and Scatter Pie Plot functions. Background map was extracted from https://www.data.gouv.fr/fr/datasets/contours-des-regions-francaises-sur-openstreetmap/and centroid data was extracted from https://www.ign.fr/reperes/centre-geographique-des-regions-metropolitaines.

### 2.5 Application of *lfb1* genotyping to clinical samples

The *lfb1* genotyping method was applied to 170 clinical samples: 110 DNA samples from humans and 60 from dogs testing positive by RT-PCR for *lfb1* and 16S rRNA, respectively (Merien et al., 2005; Waggoner et al., 2014). The human DNA samples were obtained from 60 blood samples, 17 urine samples, 4 cerebrospinal fluid samples and 29 DNA extracts from associated laboratories. The dog DNA samples were extracted from 34 blood samples, 21 urine samples and 5 kidney tissue samples.

For both humans and dogs, DNA was extracted with the QIAamp DNA mini kit (Qiagen, Germany), and PCR was performed as described by Merien et al. (2005). The CFX96 real-time PCR detection system (Bio-Rad) was used for qPCR SYBR green assays. The amplification mixture consisted of 0.4 μM primers (lfb1-F 3’-CATTCATGTTTCGAATCATTTCAAA-5’ and lfb1-R 3’-GGCCCAAGTTCCTTCTAAAAG-5’), 10 μl of SsoFast EvaGreen supermix (Bio-Rad), and 5 μl sample DNA in a total volume of 20 μl. Samples were amplified with the following program: initial denaturation at 98°C for 2 min, followed by 50 cycles of denaturation for 5 s at 98°C and annealing/elongation for 30 s at 57°C. A 331-bp fragment was amplified corresponding to the complete gene *lfb1* for *L.interrogans* serovar Icterohaemorrhagiae.

All positive PCR products were subjected to Sanger sequencing (Eurofins Scientific, Colonia, Germany and Genoscreen, Lille, France).

## 3 Results

### 3.1 *lfb1*-derived phylogeny “reference classification”

We selected a total of 834 genomes of pathogenic *Leptospira* isolates (corresponding to 227 different cgMLST clonal groups or CGs), from which *lfb1* sequences were extracted for phylogenetic analysis (**Table 1**). A single-nucleotide polymorphism (SNP) in the alignment between two sequences was considered significant for the creation of *Leptospira* species-groups (SGs).

**Figure 1:**
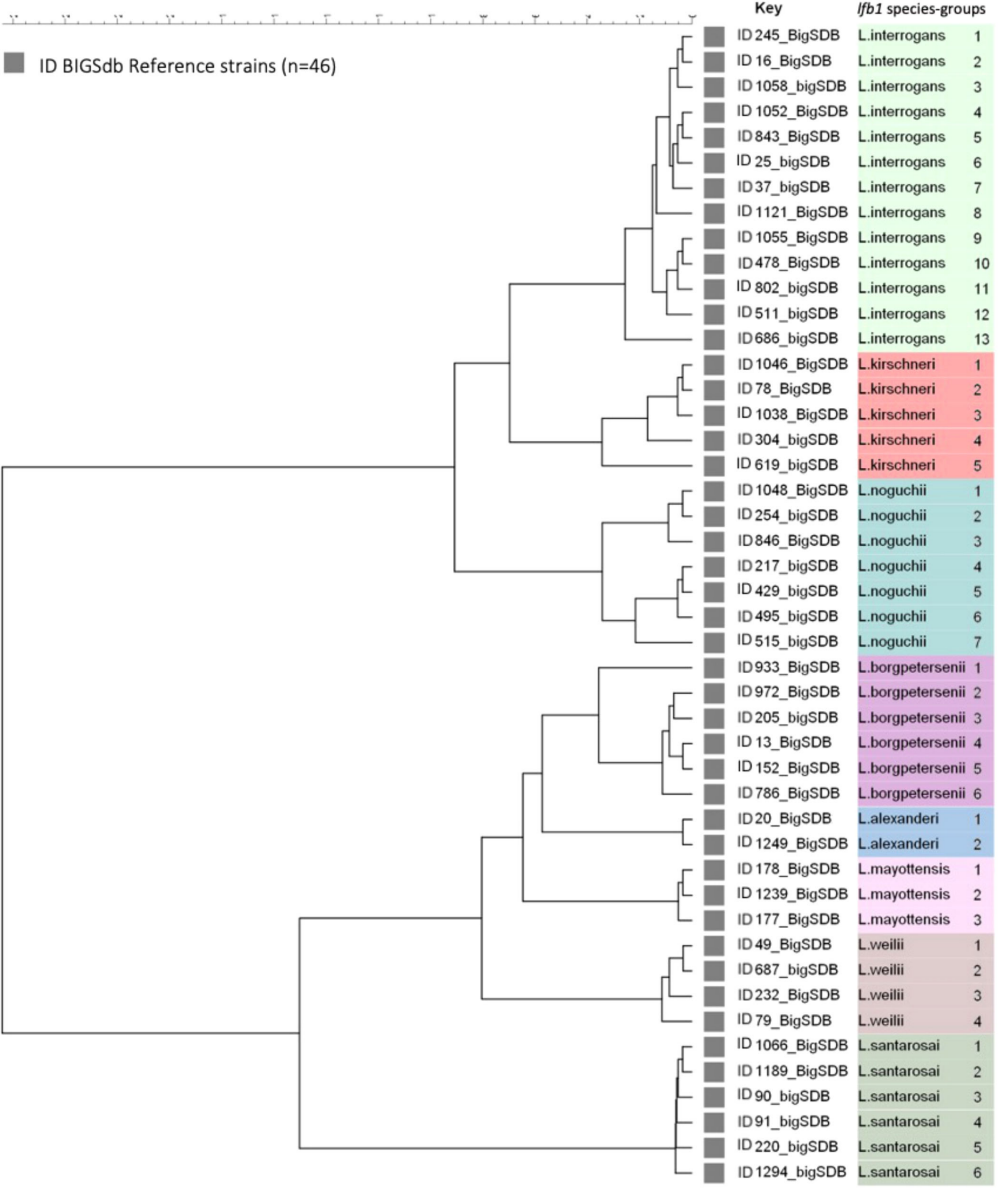
Maximum likelihood tree inferred from a *Leptospira* spp. *Ifb 1* partial gene in 46 reference strains. The ID BIGSdb accession numbers are indicated for the reference strains. Further information about the reference strains is provided in **Table 1**.

The selected genomes belong to the eight pathogenic *Leptospira* species and the analysis of a 334 bp fragment from *lfb1* identified 46 different *Leptospira* SGs, distributed as follows: 13 groups for *L. interrogans*, corresponding to 76 CGs, 5 groups for *L. kirschneri* (28 CGs), 7 groups for *L. noguchii* (21 CGs), 6 for *L. borgpetersenii* (21 CGs), 2 groups for *L. alexanderi* (2 CGs), 3 groups for *L. mayottensis* (3 CGs), 4 groups for *L. weilli* (23 CGs), and 6 groups for *L. santarosai* (53 CGs) (**Figure 1**).

### 3.2 Application of *lfb1* genotyping to clinical samples

From 2019 to 2021, we collected 110 samples from humans and 60 samples from dogs that had tested positive for leptospirosis by RT-PCR. The *lfb1* gene was sequenced from these samples. The sequences obtained and compiled with the classification data proposed above (**Figure 1**) revealed considerable genetic diversity in samples from humans and dogs, which could be subdivided into three *Leptospira* species: *L. interrogans*, *L. kirschneri* and *L. borgpetersenii*, corresponding to a total of 11 SGs. We detected *L. interrogans* in 67/110 (60.9%) of humans and in 59/60 (98.3%) of dog samples, and *L. kirschneri* in 39/110 (35.5%) of humans and 1/60 (1.7%) in dog samples. *L. borgpetersenii* was detected only in 4/110 (3.6%) of human samples.

Based on the classification established above, we detected *L. interrogans* with eight *lfb1* SGs and *L. kirschneri* with two *lfb1* SGs in humans and dogs; *L. borgpetersenii* was represented by a single *lfb1* SG found exclusively in humans. More detailed information is provided in **Table 2**.

**Table 2:** *lfb1* species-groups identification for positive human and dog samples, by year.

*lfb1* Species-group	Serogroup	Serovar	Humans *n*=110			Dogs *n*=60		
			2019	2020	2021	2019	2020	2021
*L.interrogans* SG1	Icterohaemorrhagiae	Icterohaemorrhagiae/Copenhageni	14	18	21	8	7	24
*L.interrogans* SG5	Australis Bataviae Grippotyphosa	Bratislava/Lora/Jalna/Muenchen/Fugis Bataviae Valbuzzi		1	8	2	5	8
*L.interrogans* SG10	Canicola Pomona Sejröe	Canicola Pomona/Kennewicki Medanensis			2	1		1
*L.interrogans* SG14	New group - Unknown	New group - Unknown						1
*L.interrogans* SG15	New group - Unknown	New group - Unknown		1				
*L.interrogans* SG16	New group - Unknown	New group - Unknown						2
*L.interrogans* SG17	New group - Unknown serogroup	New group - Unknown	1					
*L.interrogans* SG18	New group - Unknown serogroup	New group - Unknown	1					
*L.kirschneri* SG1	Grippotyphosa Cynopteri Pomona Mini Autumnalis Icterohaemorrhagiae	Grippotyphosa/Valbuzzi/Vanderhoedeni Unknown Pomona/Mozdok Unknown Bim Mwogolo/Sokoine	3	3	28			
*L.kirschneri* SG4	Pomona	Tsaratsovo			5			1
*L.borgpetersenii* SG1	Sejröe Ballum Javanica Mini	Sejröe Ballum/Castellonis/Kenya/Arborea/SarMini Javanica Mini/Szwajizak			4			
Total number	11	11	19	23	68	12	12	36

**Figure 2:**
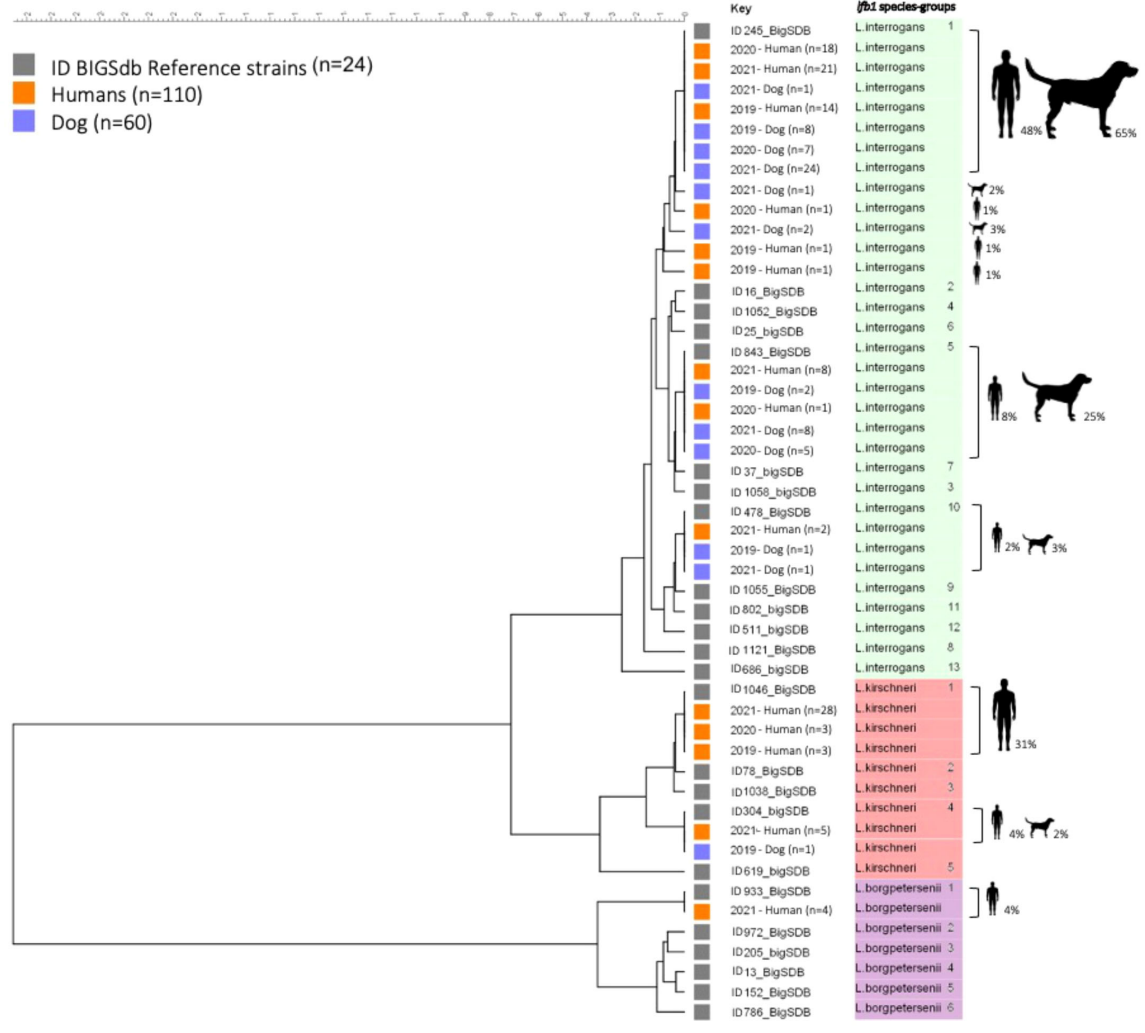
Phylogenetic tree based on *fbl 1* sequences from human and dog samples. Maximum likelihood tree inferred from *Leptospira* spp. *lfb 1* partial gene polymorphism in clinical specimens and reference strains. Gray boxes indicate reference strains, orange boxes indicate infected humans and blue boxes indicate infected dogs. The colors on the right (green, red, and purple) correspond to three different species of *Leptospira*. The ID BIGSdb numbers of the reference strains are indicated. Further information about the reference strains is provided in **Table 2**.

The *lfb1* phylogenetic analyses of the distribution *Leptospira* strains from human and dog samples are shown in **Figure 2**. In human samples, *L. interrogans* SG1 (sv Icterohaemorrhagiae/Copenhageni) was detected in 53/110 (48.2%), *L. interrogans* SG5 (sv Bratislava/Lora/Jalna/Muenchen/Bataviae) in 9/110 (8.2%), *L. interrogans* SG10 (sv Canicola/Pomona) in 2/110 (1.8%), *L. kirschneri* SG1 (sv Grippotyphosa/Valbuzzi) in 34/110 (30.9%), *L. kirschneri* SG4 (sv Tsaratsovo) in 5/110 (4.5%), and *L. borgpetersenii* SG1 (sv Sejroë/Ballum/Castellonis/Mini) in 4/110 (3.6%) samples. Three new *L. interrogans* SGs were detected in 3/110 (2.7%) samples: SG15, SG17 and SG18. In patients, the diversity SGs were retrieved in all types of biological matrices (blood, urine and CSF) with no particular tropism for an organ, such as the kidney or the brain. For example, the four CSF extracts were identified as *L. interrogans* SG1 (*n=*1), *L. interrogans* SG10 (*n=*1) and *L. kirschneri* SG1 (*n=*2).

*L. interrogans* SG1 (sv Icterohaemorrhagiae/Copenhageni) was the most frequently detected SG in dog samples, being found in 39/60 samples (65.0%), followed by *L. interrogans* SG5 (sv Bratislava/Lora/Jalna/Muenchen/Bataviae) in 15/60 (25.0%), *L. interrogans* SG10 (sv Canicola/Pomona) in 2/60 (3.3%) and *L. kirschneri* SG4 (sv Tsaratsovo) in 1/60 (1.7%). Two new *L. interrogans* SGs, SG14 and SG16, were identified in 3/60 (5.0%) samples.

### 3.3 Epidemiology of leptospirosis in both humans and dogs in France, 2019-2021

#### 3.3.1 Incidence in general human population, seasonality and serotyping

676 human cases in 2019, 450 human cases in 2020, and 708 human cases in 2021 of leptospirosis were diagnosed clinically and biologically in mainland France, corresponding to an average incidence per 100,000 inhabitants of 1.04 for 2019, 0.69 for 2020, and 1.08 for 2021 (**Supplementary Table S1**). The number of cases clearly increased from July to November in 2019 and 2021, and increased to a lesser extent over this period in 2020 (Picardeau, 2020). Detailed information is available from **Supplementary Figure S1**. For cases with positive MAT results, the most frequently identified serogroup was Icterohaemorrhagiae (34.4%). More detailed information is provided in **Supplementary Figure S2**.

#### 3.3.2 Epidemiology of positive samples identified by *lfb1* sequencing

Over the study period, the number of human specimens analyzed was highest for 2021 (68 cases, 61.8%), followed by 2020 (23 cases) and 2019 (19 cases). In dogs, the number of cases was highest in 2021 (36 cases, 60%), followed by 2020 (12 cases) and 2019 (12 cases). More detailed information is provided in **Table 3**.

**Table 3:** Epidemiological observations.

Characteristics	*n*=170		Year			Sex		Age group				Vaccine			
			2019	2020	2021	M	F	Young ≤18 years (Dogs ≤2 years)	Adult 18-65 years (Dogs 3-9 years)	Old ≥ 65 years (Dogs ≥ 10 years)	Median (range)	No	Yes	Unknown	Vaccination status
**Humans**	110	n	19	23	68	96	14	12	67	31	49 (5-84)	110	0	0	0%
		%	17.3	20.9	61.8	87.3	12.7	11	61	28		100	0	0	
**Dogs**	60	n	36	12	12	40	20	26	29	5	4 (0-13)	24	30	6	50%
		%	60	20	20	66.7	33.3	43.3	48.3	8.3		40	50	10	

The month with the largest number of notifications of leptospirosis in humans was August with an average number of 30 cases (27.3%) whereas the numbers of notifications for dogs were highest in July and September, with 11 cases in each of these months (18.3%). For human samples, a male preponderance was observed, with 96/110 (87.3%) of cases obtained from male individuals *vs*. 14/110 (12.7%) from female individuals. More than four fifths (89%) of the patients were adults and the median age was 49 years (5–84 years). A male preponderance was also observed in dogs, with 66.7% of cases (40/60) occurring in male dogs *vs*. 33.3% (20/60) in females. Just over half the dogs were adults (56.7%) and the median age was four years (0-13 years) (**Table 3**).

**Figure 3:**
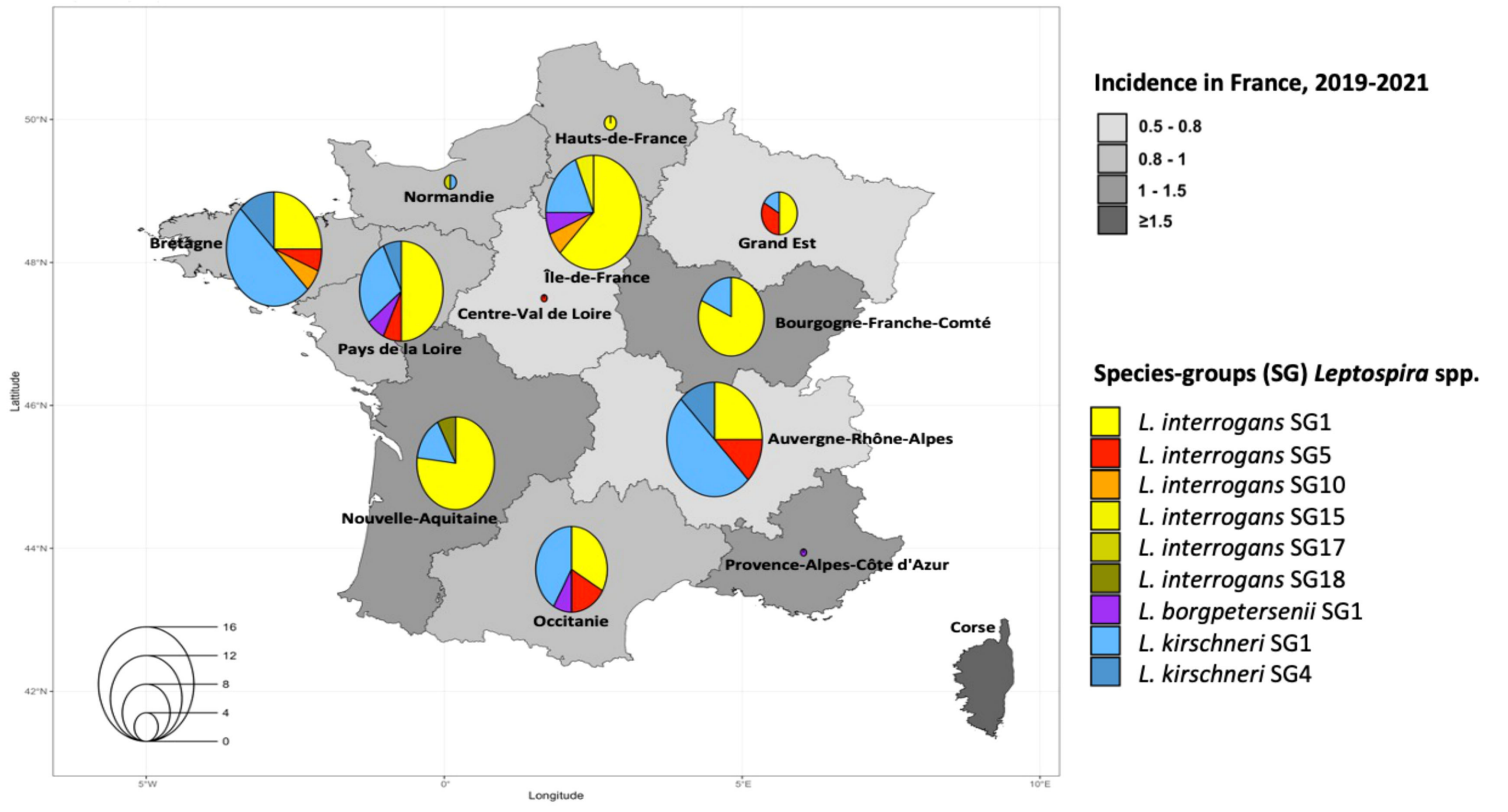
Estimated three-year mean annual human incidence of leptospirosis by region in mainland France (2019-2021). Mean annual incidence is represented as an exponential color gradient from light grey (0.5-0.8) to dark gray (≥1.5), in cases per 100,000 population. Circles indicate the distribution of the different infecting species-groups, identified in 110 humans from our study.

The predominant SG in humans was *L. interrogans* SG1 - Icterohaemorrhagiae/Copenhageni in the 13 regions of mainland France (**Figure 3**). The genotypes circulating in human cases were identified in 34 departments (an administrative area equivalent to a county) and 12 regions. The largest number of cases in a region was recorded for Auvergne-Rhône-Alpes in 2021 (Ayral et al., 2016), followed by Pays de la Loire in 2020 (Ellis, 2015), and Bourgogne-Franche-Comté in 2019 (Goarant, 2016). The genotypes in canine cases were identified in 30 departments and 10 regions, with Auvergne-Rhône-Alpes having the largest number of cases in both 2021 (Ayral et al., 2016) and 2020 (Pijnacker et al., 2016). More detailed information is provided in **Supplementary Table S2**.

The human population included in this study had not been vaccinated against *Leptospira* spp. By contrast, 50% (*n=*30/60) of the infected dogs included in this study had completed the full vaccination protocol for *Leptospira*. The dogs had mostly been vaccinated with L4 vaccines (63.3% of dogs; vaccine active against serovars Icterohaemorrhagiae, Canicola, Grippotyphosa, and Pomona), L3 vaccines (20%, serovars Icterohaemorrhagiae, Canicola and Grippotyphosa), or L2 vaccines (16.7%, serovars Icterohaemorrhagiae and Canicola). More detailed information is provided in **Supplementary Table S3**.

## 4 Discussion

### 4.1 *lfb1*-derived phylogeny “reference classification”

The *lfb1* gene is found only in pathogenic species of the P1 clade (Vincent et al., 2019). It encodes a putative adhesin of the “fibronectin-binding protein” family (Merien et al., 2000), suggesting that it may be important for virulence and/or colonization. This gene appears to have changed little during evolution, and is a potentially interesting marker for studies of the genetic diversity of pathogenic strains. Comparative analyses of the *lfb1* sequences extracted from available *Leptospira* genomes can be used to define different clusters or species-groups differing by between 1 and 74 nucleotides over a total length of 281 nucleotides. We found that 119 *lfb1* sequences from strains belonging to serovars Icterohaemorrhagiae or Copenhageni isolated from all continents and corresponding *to L. interrogans* SG1 (cgMLST 6) had identical *lfb1* sequences. A single SNP may, therefore, be sufficient to define a new species-group. However, some *lfb1* species-groups may contain several serovars or clonal groups, precluding precise identification. For example, *L. interrogans* SG2 is found in 32 genomes corresponding to different CGs (9, 10, 30, 39, 76, 77, 81 and 400) and different serovars. In this case, identification is less precise and may be a limitation of the proposed method.

The diversity of reservoir hosts for leptospirosis is an important parameter to be taken into account when considering the evolution of *Leptospira* spp. All these arguments suggest that this gene should be a robust phylogenetic marker.

### 4.2 Application of *lfb1* genotyping to clinical samples

SG diversity was greatest in the species *L. interrogans* (*n=*13), for which five new SGs were identified in human or dog samples. *L. interrogans* is isolated more frequently from both humans and animals than the other pathogenic species. The discovery of new SGs was unexpected and suggests that our knowledge of the strains responsible for disease remains incomplete. The *Ifb1* genogroups circulating in humans and dogs were identical for *L. interrogans* SG1/SG5/SG10, with a strong representation of *L. interrogans* SG1 (sv Icterohaemorrhagiae or Copenhageni), which is responsible for the most severe forms and for which the predominant reservoir in France is the rat (Boey et al., 2019).

The genogroups of species *L. kirschneri* were frequent in humans and displayed very little diversity (*L. kirschneri* SG1 and SG4) probably because the *L. kirschneri* SG1 group contains many different serovars/cgMLST, rendering high-resolution strain discrimination impossible. Similarly, *L. interrogans* SG5 and *L. borgpetersenii* SG1 may correspond to several different clonal groups or serovars. The combination of this approach with more refined tools, such as MLST and VNTR, might facilitate more precise identification in such cases. However, our previous analysis of the core genome of strains isolated from several patients (Grillova et al., 2023) showed that strains belonging to cgMLST CG64 (*n=*10/12) corresponding to *L. kirschneri* sv Grippotyphosa sg Grippotyphosa (*L. kirschneri* SG1) and to cgMLST CG72 corresponding to *L. borgpetersenii* sg Sejroe sv Sejroe predominated in France. The *L. kirschneri* SG1 genogroup was not detected in any of the dog samples, possibly due to the low level of exposure of dogs to infected environments or a lower susceptibility to *L. kirschneri* SG1 strains. Indeed, dogs with few symptoms are not presented to veterinarians and recover spontaneously. The *L. kirschneri* SG4 group was represented by only two strains isolated from a mouse in Bulgaria (identified as serovar Tsaratsovo sg Pomona) and a strain isolated from a French patient in 1990. Samples corresponding to this group were identified only in 2021, and were obtained from five human patients and one dog. The number of samples analyzed was much larger in 2021 than in 2019 and 2020, and this may have made it possible to identify less frequent genogroups.

*L. borgpetersenii* SG1 was found exclusively in samples from four patients in 2021. It was not found in dogs. It is possible that the human patients were contaminated by cattle, mice or bats, as previously suggested (Grégoire et al., 2020).

Our data indicate that leptospirosis is common in dogs in mainland France. *L. interrogans* SG1 (sv Icterohaemorrhagiae/Copenhageni) and *L. interrogans* SG5 (sv Bratislava/Lora/Jalna/Muenchen/Bataviae) predominate in our study and were clearly linked to clinical leptospirosis, whereas *L. kirschneri* infections may be associated with mild clinical symptoms or possibly be linked to asymptomatic carriage (Renaud et al., 2013; Goy-Thollot et al., 2018). The genotype distribution in dogs were consistent with the findings of previous studies in France (Renaud et al., 2013; Ayral et al., 2014; André-Fontaine and Triger, 2018; Hidalgo Friaz et al., 2023). Prevention is the best way to protect dogs from leptospirosis. It can be achieved through vaccination, avoiding contact with contaminated water and infected animals. In this study, the human patients were not vaccinated, because vaccination against leptospirosis is reserved for individuals in certain high-risk professions in France. By contrast, the leptospirosis vaccine is one of the recommended the core vaccines for dogs in France. Nevertheless, 50% of dogs included in this study were diagnosed with leptospirosis despite complete vaccination annually. Several studies have reported similar results, demonstrating the relative nature of the efficacy of current vaccines in France (Ayral et al., 2014; André-Fontaine and Triger, 2018; Bertasio et al., 2020). However, quadrivalent vaccines (L4, directed against serogroups Canicola, Icterohaemorrhagiae, Grippotyphosa and Australis) would be a more appropriate vaccine for preventing severe forms and deaths from leptospirosis (Klaasen et al., 2022; Hidalgo Friaz et al., 2023), provided that vaccination protocols are properly respected by owners and veterinarians.

### 4.3 Incidence of human of leptospirosis

The incidence of leptospirosis in humans over the three years of the study was about 1 per 100,000 inhabitants, but was lower, at only 0.69 per 100,000 inhabitants, in 2020. This decrease is probably related to the COVID-19 pandemic, during which recreational activities were limited and the number of imported cases was decreased by travel restrictions. Similarly, little seasonality was observed in 2020.

Incidence was highest in regions with livestock production, high rainfall, and large rivers. However, some fluctuation was observed from year to year and the data may not necessarily reflect the situation on the ground at a particular time point. For example, people may become infected during their summer vacation but are not diagnosed until they return to their home region.

The French Ministry of Health plans to declare leptospirosis to be a notifiable disease in the near future. This initiative will improve the follow-up of cases and provide associated epidemiological data (number of cases, disease severity, site of exposure, mode of contamination). The FNRC will play an important role in confirming the biological diagnosis and tracking the serogroups/genogroups involved in these infections. The follow-up of serogroups based on MAT shows that Icterohaemorrhagiae is the most frequently encountered serogroup, consistent with the results of *Ifb1* genotyping. However, very little information is available for the other serogroups. The regional distribution of origin of samples and of the *lfb1* genogroups to identify in mainland France appears to be generally homogeneous for human infection, but not for dogs, for which samples were mostly obtained in the Rhone-Alpes region, in which the LAV laboratory location. More regular surveillance of canine cases in different French regions over a period of several years would be useful as it would provide a more precise idea of the *Leptospira* genotypes circulating in this species, and would make it possible to establish the epidemiological basis of transmission between humans and dogs. Such surveillance would also make it possible to update preventive measures in humans and animals.

### 4.4 Conclusion

We provide here the first global description of the *Leptospira* strains responsible for acute infections in humans and dogs in mainland France. We demonstrate that polymorphism of the *lfb1* gene is a robust method to provide rapid identification using biological samples. This tool has enabled us to also identify five *lfb1 L. interrogans* species-groups never before described. The availability of more precise epidemiological data in the future should facilitate the identification of sources of animal and environmental contamination, making it possible to establish public health control interventions.

## Data availability statement

The datasets presented in this study can be found in online repositories. The names of the repository/repositories and accession number(s) can be found below: https://www.ncbi.nlm.nih.gov/genbank/, OR101259 - OR101474.

## Author contributions

MG-L: Data curation and analyses. CL, EH: Data curation. AS, ST-P: Samples for diagnostic. FA: Data analysis and review. PB contributed to conception and design of the study and data analyses. MG-L, ZD, MP, PB contributed to the writing and editing of manuscript. All authors contributed to manuscript revision, read, and approved the submitted version.
