# Supplementary material for: Genetic diversity of Leptospira strains circulating in humans and dogs in France in 2019-2021
Source: Front Cell Infect Microbiol. 2023 Aug 17;13:1236866. doi: 10.3389/fcimb.2023.1236866 (PMC10469827; doi:10.3389/fcimb.2023.1236866)
Supplement: Supplementary file 1 [file Table_1.docx]

Table S1: Incidence of human leptospirosis per 100,000 inhabitants in the 13 regions of mainland France, 2019-2021.

| Region | *Départements* | 2019 | | | 2020 | | | 2021 | | |  |
| --- | --- | --- | --- | --- | --- | --- | --- | --- | --- | --- | --- |
|  | Population (hab.) | Population (inhabitants) | Number of cases | Incidence | Population (inhabitants) | Number of cases | Incidence | Population (inhabitants) | Number of cases | Incidence |  |
| Île-de-France | 75 77 78 91 92 93 94 95 | 12,262,544 | 153 | 1.25 | 12,308,593 | 85 | 0.69 | 12,348,605 | 127 | 1.03 |  |
| Auvergne-Rhône-Alpes | 01 03 07 15 26 38 42 43 63 69 73 74 | 8,042,936 | 43 | 0.53 | 8,082,099 | 30 | 0.37 | 8,113,805 | 98 | 1.21 |  |
| Hauts-de-France | 02 59 60 62 80 | 6,004,947 | 51 | 0.85 | 6,001,668 | 34 | 0.57 | 5,994,428 | 61 | 1.02 |  |
| Nouvelle-Aquitaine | 16 17 19 23 24 33 40 47 64 79 86 87 | 6,010,289 | 76 | 1.26 | 6,033,690 | 88 | 1.46 | 6,058,437 | 104 | 1.72 |  |
| Occitanie | 09 11 12 30 31 32 34 46 48 65 66 81 82 | 5,933,185 | 59 | 0.99 | 5,972,810 | 34 | 0.57 | 6,013,289 | 55 | 0.91 |  |
| Grand Est | 08 10 51 52 54 55 57 67 68 88 | 5,556,219 | 39 | 0.70 | 5,554,478 | 28 | 0.50 | 5,546,553 | 35 | 0.63 |  |
| Provence-Alpes-Côte d'Azur | 04 05 06 13 83 84 | 5,081,101 | 108 | 2.13 | 5,098,994 | 19 | 0.37 | 5,116,360 | 43 | 0.84 |  |
| Pays de la Loire | 44 49 53 72 85 | 3,806,461 | 27 | 0.71 | 3,827,922 | 36 | 0.94 | 3,849,977 | 30 | 0.78 |  |
| Normandie | 14 27 50 61 76 | 3,325,032 | 35 | 1.05 | 3,319,638 | 23 | 0.69 | 3,313,398 | 36 | 1.09 |  |
| Bretagne | 22 29 35 56 | 3,354,854 | 19 | 0.57 | 3,370,113 | 32 | 0.95 | 3,386,415 | 47 | 1.39 |  |
| Bourgogne-Franche-Comté | 21 25 39 58 70 71 89 90 | 2,805,580 | 40 | 1.43 | 2,800,016 | 20 | 0.71 | 2,791,809 | 35 | 1.25 |  |
| Centre-Val de Loire | 18 28 36 37 41 45 | 2,573,180 | 16 | 0.62 | 2,570,734 | 13 | 0.51 | 2,567,768 | 29 | 1.13 |  |
| Corse | 2A 2B | 340,440 | 10 | 2.94 | 343,634 | 6 | 1.75 | 346,610 | 8 | 2.31 |  |
| TOTAL | | 65,096,768 | 676 | 1.04 | 65,284,389 | 450 | 0.69 | 65,447,454 | 708 | 1.08 |  |

Table S2: Number of sample genotype patterns per *lfb1* sequence by region and year.

| Region, by year | Humans | | | | Dogs | | | |
| --- | --- | --- | --- | --- | --- | --- | --- | --- |
|  | *n*=110 | 2019 | 2020 | 2021 | *n*=60 | 2019 | 2020 | 2021 |
| Auvergne-Rhône-Alpes | 16 | 0 | 1 | 15 | 22 | 3 | 4 | 15 |
| Bourgogne-Franche-Comté | 11 | 6 | 2 | 3 | 9 | 3 | 1 | 5 |
| Bretagne | 16 | 1 | 1 | 14 | 1 | 0 | 0 | 1 |
| Centre-Val de Loire | 1 | 0 | 0 | 1 | 2 | 0 | 1 | 1 |
| Corse | 0 | 0 | 0 | 0 | 0 | 0 | 0 | 0 |
| Grand Est | 6 | 1 | 1 | 4 | 4 | 0 | 1 | 3 |
| Hauts-de-France | 2 | 2 | 0 | 0 | 0 | 0 | 0 | 0 |
| Île-de-France | 16 | 3 | 4 | 9 | 2 | 0 | 1 | 1 |
| Normandie | 2 | 2 | 0 | 0 | 0 | 0 | 0 | 0 |
| Nouvelle-Aquitaine | 13 | 1 | 6 | 6 | 12 | 3 | 1 | 8 |
| Occitanie | 12 | 1 | 0 | 11 | 3 | 1 | 1 | 1 |
| Pays de la Loire | 14 | 2 | 8 | 4 | 3 | 0 | 2 | 1 |
| Provence-Alpes-Côte d'Azur | 1 | 0 | 0 | 1 | 2 | 2 | 0 | 0 |
| Total | 110 | 19 | 23 | 68 | 60 | 12 | 12 | 36 |

Table S3: List of clinical samples, including sample identification, species (human or dog), date of sample collection (day, month and year), sex, age, sample type (blood, urine, kidney, CSF, DNA), location (zip code and region), clinical signs, contact with animals or water, antibiotic treatment, vaccination (vaccination status and type of vaccine used) and molecular results for *Leptospira* spp.

| N | ID sample | Species | Date of sample collection | Sample type | Sex | Age | Zip Code | Region of France | Vaccination | Identification Leptospira spp. |
| --- | --- | --- | --- | --- | --- | --- | --- | --- | --- | --- |
| 1 | 201900068 | Human | 3/1/2019 | Blood | M | 84 | 54 | Grand Est | Not | L. interrogans SG1 |
| 2 | 201900767 | Human | 2/4/2019 | Blood | M | 29 | 25 | Bourgogne-Franche-Comté | Not | L. interrogans SG1 |
| 3 | 201900961 | Human | 30/4/2019 | Urine | M | 69 | 90 | Bourgogne-Franche-Comté | Not | L. interrogans SG1 |
| 4 | 201901166 | Human | 31/5/2019 | Blood | M | 74 | 33 | Nouvelle-Aquitaine | Not | L. interrogans SG18* |
| 5 | 201901168 | Human | 29/5/2019 | Urine | M | 49 | 14 | Normandie | Not | L. interrogans SG17* |
| 6 | 201901647 | Human | 25/7/2019 | Blood | M | 69 | 30 | Occitanie | Not | L. interrogans SG1 |
| 7 | 201901703 | Human | 31/7/2019 | Blood | M | 22 | 21 | Bourgogne-Franche-Comté | Not | L. interrogans SG1 |
| 8 | 201901710 | Human | 1/8/2019 | Blood | M | 21 | 25 | Bourgogne-Franche-Comté | Not | L. interrogans SG1 |
| 9 | 201901730 | Human | 2/8/2019 | Blood | M | 21 | 25 | Bourgogne-Franche-Comté | Not | L. interrogans SG1 |
| 10 | 201901772 | Human | 3/8/2019 | Urine | M | 33 | 60 | Hauts-de-France | Not | L. interrogans SG1 |
| 11 | 201901794 | Human | 1/8/2019 | Urine | M | 15 | 60 | Hauts-de-France | Not | L. interrogans SG1 |
| 12 | 201901814 | Human | 9/8/2019 | Blood | M | 48 | 49 | Pays de la Loire | Not | L.kirschneri SG1 |
| 13 | 201902037 | Human | 2/9/2019 | Blood | M | 16 | 25 | Bourgogne-Franche-Comté | Not | L. interrogans SG1 |
| 14 | 201902089 | Human | 4/9/2019 | Blood | M | 17 | 14 | Normandie | Not | L.kirschneri SG1 |
| 15 | 201902799 | Human | 15/11/2019 | Blood | M | 60 | 49 | Pays de la Loire | Not | L. interrogans SG1 |
| 16 | 201902845-46 | Human | 21/11/2019 | Blood | M | 61 | 91 | Île-de-France | Not | L. interrogans SG1 |
| 17 | 201902864 | Human | 22/11/2019 | Blood | F | 43 | 35 | Bretagne | Not | L.kirschneri SG1 |
| 18 | 201902915 | Human | 29/11/2019 | Blood | M | 41 | 92 | Île-de-France | Not | L. interrogans SG1 |
| 19 | 201903031-32 | Human | 11/12/2019 | Blood | M | 72 | 75 | Île-de-France | Not | L. interrogans SG1 |
| 20 | 202000120 | Human | 9/1/2020 | Blood | M | 54 | 69 | Auvergne-Rhône-Alpes | Not | L. interrogans SG1 |
| 21 | 202000244 | Human | 30/1/2020 | Blood | F | 59 | 85 | Pays de la Loire | Not | L. interrogans SG1 |
| 22 | 202000340 | Human | 13/2/2020 | Blood | M | 43 | 85 | Pays de la Loire | Not | L. interrogans SG1 |
| 23 | 202000405 | Human | 26/2/2020 | Blood | F | 70 | 85 | Pays de la Loire | Not | L. interrogans SG1 |
| 24 | 202000489 | Human | 18/3/2020 | Blood | M | 41 | 85 | Pays de la Loire | Not | L.kirschneri SG1 |
| 25 | 202000627 | Human | 28/4/2020 | Blood | F | 65 | 33 | Nouvelle-Aquitaine | Not | L. interrogans SG1 |
| 26 | 202000942 | Human | 17/6/2020 | Blood | M | 67 | 40 | Nouvelle-Aquitaine | Not | L. interrogans SG1 |
| 27 | 202000963 | Human | 23/6/2020 | Blood | M | 43 | 75 | Île-de-France | Not | L. interrogans SG15* |
| 28 | 202001077 | Human | 7/7/2020 | Urine | M | 32 | 92 | Île-de-France | Not | L. interrogans SG1 |
| 29 | 202001149 | Human | 13/7/2020 | Blood | M | 20 | 64 | Nouvelle-Aquitaine | Not | L. interrogans SG1 |
| 30 | 202001249 | Human | 28/7/2020 | Blood | M | 69 | 85 | Pays de la Loire | Not | L. interrogans SG1 |
| 31 | 202001313 | Human | 6/8/2020 | Blood | M | 74 | 21 | Bourgogne-Franche-Comté | Not | L. interrogans SG1 |
| 32 | 202001360 | Human | 14/8/2020 | Blood | F | 18 | 75 | Île-de-France | Not | L. interrogans SG1 |
| 33 | 202201399 | Human | 11/11/2020 | DNA | M | 58 | 22 | Bretagne | Not | L. interrogans SG1 |
| 34 | 202001400 | Human | 20/8/2020 | Blood | M | 37 | 21 | Bourgogne-Franche-Comté | Not | L. interrogans SG1 |
| 35 | 202001454 | Human | 28/8/2020 | Urine | M | 25 | 57 | Grand Est | Not | L. interrogans SG1 |
| 36 | 202001456 | Human | 27/8/2020 | Blood | M | 63 | 86 | Nouvelle-Aquitaine | Not | L.kirschneri SG1 |
| 37 | 202001596 | Human | 15/9/2020 | Urine | M | 73 | 85 | Pays de la Loire | Not | L. interrogans SG1 |
| 38 | 202001659 | Human | 26/9/2020 | Blood | M | 52 | 86 | Nouvelle-Aquitaine | Not | L. interrogans SG1 |
| 39 | 202001668 | Human | 29/9/2020 | Urine | M | 39 | 75 | Île-de-France | Not | L. interrogans SG1 |
| 40 | 202001699 | Human | 1/10/2020 | Blood | M | 72 | 85 | Pays de la Loire | Not | L. interrogans SG5 |
| 41 | 202002051 | Human | 19/11/2020 | Blood | M | 55 | 64 | Nouvelle-Aquitaine | Not | L. interrogans SG1 |
| 42 | 202002154 | Human | 28/11/2020 | Blood | M | 65 | 85 | Pays de la Loire | Not | L.kirschneri SG1 |
| 43 | 202100047 | Human | 7/1/2021 | CSF | M | 71 | 86 | Nouvelle-Aquitaine | Not | L. interrogans SG1 |
| 44 | 202100139 | Human | 23/1/2021 | Blood | M | 47 | 25 | Bourgogne-Franche-Comté | Not | L.kirschneri SG1 |
| 45 | 202100474 | Human | 25/2/2021 | Blood | M | 49 | 85 | Pays de la Loire | Not | L.borgpetersenii SG1 |
| 46 | 202100989 | Human | 30/4/2021 | Blood | M | 52 | 75 | Île-de-France | Not | L. interrogans SG1 |
| 47 | 202101165 | Human | 28/5/2021 | Blood | M | 48 | 29 | Bretagne | Not | L.kirschneri SG1 |
| 48 | 202101402 | Human | 28/6/2021 | Blood | M | 32 | 86 | Nouvelle-Aquitaine | Not | L. interrogans SG1 |
| 49 | 202101443 | Human | 5/7/2021 | Urine | F | 30 | 75 | Île-de-France | Not | L. interrogans SG1 |
| 50 | 202101504 | Human | 10/7/2021 | Blood | M | 67 | 30 | Occitanie | Not | L.borgpetersenii SG1 |
| 51 | 202101578 | Human | 20/7/2021 | Blood | M | 72 | 85 | Pays de la Loire | Not | L. interrogans SG1 |
| 52 | 202101618 | Human | 27/7/2021 | Urine | M | 36 | 94 | Île-de-France | Not | L. interrogans SG1 |
| 53 | 202101794 | Human | 10/8/2021 | Blood | M | 65 | 85 | Pays de la Loire | Not | L.kirschneri SG1 |
| 54 | 202101815 | Human | 6/8/2021 | Blood | M | 25 | 69 | Auvergne-Rhône-Alpes | Not | L.kirschneri SG1 |
| 55 | 202101839 | Human | 16/8/2021 | CSF | M | 27 | 75 | Île-de-France | Not | L. interrogans SG10 |
| 56 | 202101854 | Human | 17/8/2021 | CSF | F | 42 | 73 | Auvergne-Rhône-Alpes | Not | L.kirschneri SG1 |
| 57 | 202101939 | Human | 26/8/2021 | Blood | F | 19 | 95 | Île-de-France | Not | L.borgpetersenii SG1 |
| 58 | 202101945 | Human | 26/8/2021 | Blood | M | 68 | 57 | Grand Est | Not | L. interrogans SG1 |
| 59 | 202101946 | Human | 26/8/2021 | Blood | M | 83 | 22 | Bretagne | Not | L. interrogans SG1 |
| 60 | 202101948 | Human | 26/8/2021 | Blood | M | 10 | 83 | Provence-Alpes-Côte d'Azur | Not | L.borgpetersenii SG1 |
| 61 | 202101949 | Human | 26/8/2021 | Urine | M | 42 | 33 | Nouvelle-Aquitaine | Not | L. interrogans SG1 |
| 62 | 202101952 | Human | 26/8/2021 | Blood | M | 60 | 19 | Nouvelle-Aquitaine | Not | L. interrogans SG1 |
| 63 | 202101954 | Human | 26/8/2021 | Blood | F | 24 | 77 | Île-de-France | Not | L.kirschneri SG1 |
| 64 | 202101956 | Human | 26/8/2021 | Blood | M | 48 | 67 | Grand Est | Not | L.kirschneri SG1 |
| 65 | 202101992 | Human | 30/8/2021 | Urine | M | 74 | 51 | Grand Est | Not | L. interrogans SG5 |
| 66 | 202102131 | Human | 6/9/2021 | Blood | M | 15 | 9 | Occitanie | Not | L.kirschneri SG1 |
| 67 | 202102169 | Human | 1/9/2021 | Blood | M | 41 | 9 | Occitanie | Not | L.kirschneri SG1 |
| 68 | 202102170 | Human | 10/9/2021 | Blood | F | 11 | 9 | Occitanie | Not | L.kirschneri SG1 |
| 69 | 202102198 | Human | 8/9/2021 | Urine | M | 40 | 38 | Auvergne-Rhône-Alpes | Not | L.kirschneri SG1 |
| 70 | 202102199 | Human | 8/9/2021 | CSF | M | 48 | 35 | Bretagne | Not | L.kirschneri SG1 |
| 71 | 202102200 | Human | 30/8/2021 | Blood | M | 11 | 38 | Auvergne-Rhône-Alpes | Not | L.kirschneri SG1 |
| 72 | 202102202 | Human | 25/8/2021 | Blood | M | 38 | 95 | Île-de-France | Not | L. interrogans SG1 |
| 73 | 202102203 | Human | 30/8/2021 | Blood | F | 50 | 95 | Île-de-France | Not | L.kirschneri SG1 |
| 74 | 202102216 | Human | 21/9/2021 | Urine | M | 67 | 57 | Grand Est | Not | L. interrogans SG5 |
| 75 | 202102229 | Human | 24/9/2021 | Urine | M | 78 | 85 | Pays de la Loire | Not | L.kirschneri SG4 |
| 76 | 202102261 | Human | 15/9/2021 | DNA | M | 16 | 69 | Auvergne-Rhône-Alpes | Not | L.kirschneri SG1 |
| 77 | 202102317 | Human | 6/10/2021 | DNA | M | 29 | 69 | Auvergne-Rhône-Alpes | Not | L. interrogans SG1 |
| 78 | 202102370 | Human | 12/10/2021 | Blood | M | 71 | 40 | Nouvelle-Aquitaine | Not | L.kirschneri SG1 |
| 79 | 202102376 | Human | 6/10/2021 | Urine | M | 62 | 56 | Bretagne | Not | L.kirschneri SG1 |
| 80 | 202102378 | Human | 16/10/2021 | Blood | M | 72 | 77 | Île-de-France | Not | L.kirschneri SG1 |
| 81 | 202102398 | Human | 16/10/2021 | Blood | M | 44 | 86 | Nouvelle-Aquitaine | Not | L. interrogans SG1 |
| 82 | 202102400 | Human | 8/10/2021 | Blood | M | 62 | 41 | Centre-Val de Loire | Not | L. interrogans SG5 |
| 83 | 202102425 | Human | 18/10/2021 | Urine | M | 44 | 21 | Bourgogne-Franche-Comté | Not | L. interrogans SG1 |
| 84 | 202102462 | Human | 21/10/2021 | Blood | M | 70 | 25 | Bourgogne-Franche-Comté | Not | L.kirschneri SG1 |
| 85 | 202102499 | Human | 30/7/2021 | DNA | M | ND | 69 | Auvergne-Rhône-Alpes | Not | L.kirschneri SG4 |
| 86 | 202102501 | Human | 19/3/2021 | DNA | M | 74 | 69 | Auvergne-Rhône-Alpes | Not | L. interrogans SG1 |
| 87 | 202102505 | Human | 17/9/2021 | DNA | M | ND | 69 | Auvergne-Rhône-Alpes | Not | L. interrogans SG5 |
| 88 | 202102512 | Human | 17/9/2021 | DNA | M | ND | 69 | Auvergne-Rhône-Alpes | Not | L.kirschneri SG1 |
| 89 | 202102514 | Human | 29/6/2021 | DNA | M | ND | 69 | Auvergne-Rhône-Alpes | Not | L. interrogans SG1 |
| 90 | 202102521 | Human | 8/9/2021 | DNA | F | ND | 69 | Auvergne-Rhône-Alpes | Not | L.kirschneri SG1 |
| 91 | 202102523 | Human | 29/10/2021 | DNA | M | ND | 69 | Auvergne-Rhône-Alpes | Not | L. interrogans SG5 |
| 92 | 202102525 | Human | 2/9/2021 | DNA | M | ND | 69 | Auvergne-Rhône-Alpes | Not | L.kirschneri SG4 |
| 93 | 202102528 | Human | 9/8/2021 | DNA | F | ND | 69 | Auvergne-Rhône-Alpes | Not | L.kirschneri SG1 |
| 94 | 202102662 | Human | 18/11/2021 | DNA | M | 77 | 31 | Occitanie | Not | L. interrogans SG5 |
| 95 | 202102663 | Human | 29/10/2021 | DNA | M | 47 | 31 | Occitanie | Not | L.kirschneri SG1 |
| 96 | 202102664 | Human | 29/10/2021 | DNA | F | 62 | 31 | Occitanie | Not | L.kirschneri SG1 |
| 97 | 202102666 | Human | 29/10/2021 | DNA | M | 38 | 31 | Occitanie | Not | L. interrogans SG1 |
| 98 | 202102667 | Human | 29/10/2021 | DNA | M | 57 | 31 | Occitanie | Not | L. interrogans SG1 |
| 99 | 202102669 | Human | 29/10/2021 | DNA | M | 55 | 31 | Occitanie | Not | L. interrogans SG1 |
| 100 | 202102674 | Human | 18/11/2021 | DNA | M | 39 | 31 | Occitanie | Not | L. interrogans SG5 |
| 101 | 202201383 | Human | 13/7/2021 | DNA | M | 24 | 35 | Bretagne | Not | L.kirschneri SG1 |
| 102 | 202201385 | Human | 14/7/2021 | DNA | M | 54 | 35 | Bretagne | Not | L. interrogans SG1 |
| 103 | 202201386 | Human | 7/8/2021 | DNA | M | 64 | 22 | Bretagne | Not | L.kirschneri SG1 |
| 104 | 202201387 | Human | 23/5/2021 | DNA | M | 62 | 22 | Bretagne | Not | L.kirschneri SG4 |
| 105 | 202201389 | Human | 9/8/2021 | DNA | M | 16 | 22 | Bretagne | Not | L.kirschneri SG1 |
| 106 | 202201390 | Human | 4/9/2021 | DNA | M | 27 | 35 | Bretagne | Not | L. interrogans SG10 |
| 107 | 202201391 | Human | 10/9/2021 | DNA | M | 36 | 22 | Bretagne | Not | L. interrogans SG5 |
| 108 | 202201392 | Human | 3/10/2021 | DNA | M | 79 | 22 | Bretagne | Not | L.kirschneri SG4 |
| 109 | 202201396 | Human | 31/7/2021 | DNA | M | 16 | 35 | Bretagne | Not | L. interrogans SG1 |
| 110 | 202201397 | Human | 3/8/2021 | DNA | M | 49 | 35 | Bretagne | Not | L.kirschneri SG1 |
| 111 | 2019-3280D | Dog | 23/1/2019 | Blood | M | 7 | 84 | Provence-Alpes-Côte d'Azur | Not | L. interrogans SG1 |
| 112 | 2019-3283D | Dog | 25/1/2019 | Blood | F | 1 | 89 | Bourgogne-Franche-Comté | Yes - L4 | L. kirschneri SG4 |
| 113 | 2019-3357D | Dog | 3/4/2019 | Blood | M | 8 | 71 | Bourgogne-Franche-Comté | Not | L. interrogans SG1 |
| 114 | 2019-3373D | Dog | 9/4/2019 | Blood | F | 6 | 69 | Auvergne-Rhône-Alpes | Not | L. interrogans SG1 |
| 115 | 2019-3404D | Dog | 23/4/2019 | Urine | M | 7 | 71 | Bourgogne-Franche-Comté | Yes - L2 | L. interrogans SG1 |
| 116 | 2019-3590D | Dog | 3/6/2019 | Blood | M | 3 | 84 | Provence-Alpes-Côte d'Azur | Not | L. interrogans SG1 |
| 117 | 2019-3640D | Dog | 25/6/2019 | Blood | F | <1 | 1 | Auvergne-Rhône-Alpes | Yes - L3 | L. interrogans SG1 |
| 118 | 2019-3735D | Dog | 30/7/2019 | Urine | F | 1 | 24 | Nouvelle-Aquitaine | Yes - L4 | L. interrogans SG5 |
| 119 | 2019-3745D | Dog | 12/8/2019 | Blood | M | 1 | 69 | Auvergne-Rhône-Alpes | Yes - L4 | L. interrogans SG1 |
| 120 | 2019-3825D | Dog | 9/9/2019 | Urine | F | 7 | 31 | Occitanie | Not | L. interrogans SG10 |
| 121 | 2019-3827D | Dog | 9/9/2019 | Blood | M | <1 | 24 | Nouvelle-Aquitaine | Not | L. interrogans SG1 |
| 122 | 2019-3850D | Dog | 30/9/2019 | Urine | M | 10 | 64 | Nouvelle-Aquitaine | Not | L. interrogans SG5 |
| 123 | 2020-226D | Dog | 24/1/2020 | Urine | M | <1 | 44 | Pays de la Loire | Not | L. interrogans SG5 |
| 124 | 2020-1100D | Dog | 4/6/2020 | Blood | M | 6 | 68 | Grand Est | Yes - L2 | L. interrogans SG5 |
| 125 | 2020-1124D | Dog | 9/6/2020 | Kidney | F | 7 | 38 | Auvergne-Rhône-Alpes | Yes - L4 | L. interrogans SG1 |
| 126 | 2020-1274D | Dog | 24/6/2020 | Blood | M | <1 | 71 | Bourgogne-Franche-Comté | ND | L. interrogans SG1 |
| 127 | 2020-1329D | Dog | 29/6/2020 | Blood | M | <1 | 44 | Pays de la Loire | Not | L. interrogans SG1 |
| 128 | 2020-1655D | Dog | 12/8/2020 | Urine | F | 7 | 26 | Auvergne-Rhône-Alpes | Yes - L4 | L. interrogans SG1 |
| 129 | 2020-1751D | Dog | 3/9/2020 | Blood | M | 4 | 77 | Île-de-France | Yes - L4 | L. interrogans SG5 |
| 130 | 2020-1784D | Dog | 8/9/2020 | Blood | M | <1 | 31 | Occitanie | Not | L. interrogans SG1 |
| 131 | 2020-2129D | Dog | 12/10/2020 | Urine | M | <1 | 17 | Nouvelle-Aquitaine | Not | L. interrogans SG1 |
| 132 | 2020-2417D | Dog | 13/11/2020 | Blood | F | 13 | 74 | Auvergne-Rhône-Alpes | Yes - L3 | L. interrogans SG5 |
| 133 | 2020-2572D | Dog | 26/11/2020 | Blood | M | <1 | 1 | Auvergne-Rhône-Alpes | Yes - L4 | L. interrogans SG5 |
| 134 | 2020-2874D | Dog | 23/12/2020 | Blood | F | <1 | 18 | Centre-Val de Loire | Yes - L4 | L. interrogans SG1 |
| 135 | 2021-50D | Dog | 8/1/2021 | Blood | M | 1 | 18 | Centre-Val de Loire | Not | L. interrogans SG1 |
| 136 | 2021-51D | Dog | 8/1/2021 | Blood | F | 1 | 1 | Auvergne-Rhône-Alpes | Not | L. interrogans SG5 |
| 137 | 2021-281D | Dog | 12/4/2021 | Blood | M | 7 | 74 | Auvergne-Rhône-Alpes | Not | L. interrogans SG1 |
| 138 | 2021-282D | Dog | 12/4/2021 | Blood | F | <1 | 17 | Nouvelle-Aquitaine | Yes - L4 | L. interrogans SG1 |
| 139 | 2021-382D | Dog | 12/4/2021 | Blood | M | 4 | 31 | Occitanie | ND | L. interrogans SG5 |
| 140 | 2021-645D | Dog | 24/4/2021 | Blood | F | <1 | 24 | Nouvelle-Aquitaine | Not | L. interrogans SG1 |
| 141 | 2021-708D | Dog | 24/4/2021 | Urine | M | 1 | 64 | Nouvelle-Aquitaine | Not | L. interrogans SG1 |
| 142 | 2021-1286D | Dog | 20/5/2021 | Urine | M | 8 | 69 | Auvergne-Rhône-Alpes | Yes - L4 | L. interrogans SG5 |
| 143 | 2021-1420D | Dog | 27/5/2021 | Blood | M | 4 | 79 | Nouvelle-Aquitaine | Yes - L3 | L. interrogans SG1 |
| 144 | 2021-1813D | Dog | 24/6/2021 | Urine | M | 2 | 67 | Grand Est | ND | L. interrogans SG1 |
| 145 | 2021-1955D | Dog | 2/7/2021 | Urine | M | 4 | 69 | Auvergne-Rhône-Alpes | Yes - L3 | L. interrogans SG1 |
| 146 | 2021-2066D | Dog | 8/7/2021 | Urine | M | 11 | 38 | Auvergne-Rhône-Alpes | Not | L. interrogans SG5 |
| 147 | 2021-2120D | Dog | 15/7/2021 | Blood | M | 10 | 71 | Bourgogne-Franche-Comté | Not | L. interrogans SG5 |
| 148 | 2021-2171D | Dog | 19/7/2021 | Blood | M | 9 | 33 | Nouvelle-Aquitaine | Not | L. interrogans SG5 |
| 149 | 2021-2175D | Dog | 19/7/2021 | Urine | F | 7 | 1 | Auvergne-Rhône-Alpes | Yes - L2 | L. interrogans SG1 |
| 150 | 2021-2235D | Dog | 23/7/2021 | Blood | M | 5 | 71 | Bourgogne-Franche-Comté | Not | L. interrogans SG1 |
| 151 | 2021-2240D | Dog | 23/7/2021 | Kidney | F | <1 | 53 | Pays de la Loire | Yes - L4 | L. interrogans SG1 |
| 152 | 2021-2304D | Dog | 29/7/2021 | Blood | M | 10 | 38 | Auvergne-Rhône-Alpes | Yes - L4 | L. interrogans SG5 |
| 153 | 2021-2315D | Dog | 29/7/2021 | Urine | M | 3 | 78 | Île-de-France | Yes - L4 | L. interrogans SG1 |
| 154 | 2021-2318D | Dog | 29/7/2021 | Kidney | F | <1 | 57 | Grand Est | Yes - L4 | L. interrogans SG1 |
| 155 | 2021-2586D | Dog | 26/8/2021 | Blood | M | <1 | 88 | Grand Est | Yes - L4 | L. interrogans SG1 |
| 156 | 2021-2596D | Dog | 26/8/2021 | Blood | M | 8 | 73 | Auvergne-Rhône-Alpes | Yes - L2 | L. interrogans SG1 |
| 157 | 2021-2703D | Dog | 30/8/2021 | Urine | F | 5 | 47 | Nouvelle-Aquitaine | Yes - L4 | L. interrogans SG10 |
| 158 | 2021-2710D | Dog | 2/9/2021 | Blood | F | 6 | 69 | Auvergne-Rhône-Alpes | ND | L. interrogans SG1 |
| 159 | 2021-2712D | Dog | 2/9/2021 | Urine | M | 7 | 58 | Bourgogne-Franche-Comté | Yes - L4 | L. interrogans SG1 |
| 160 | 2021-2835D | Dog | 8/9/2021 | Urine | M | 4 | 38 | Auvergne-Rhône-Alpes | Not | L. interrogans SG1 |
| 161 | 2021-3008D | Dog | 16/9/2021 | Urine | M | <1 | 38 | Auvergne-Rhône-Alpes | Yes - L2 | L. interrogans SG1 |
| 162 | 2021-3056D | Dog | 20/9/2021 | Blood | M | 4 | 42 | Auvergne-Rhône-Alpes | Not | L. interrogans SG5 |
| 163 | 2021-3165D | Dog | 27/9/2021 | Kidney | M | <1 | 40 | Nouvelle-Aquitaine | Not | L. interrogans SG1 |
| 164 | 2021-3455D | Dog | 15/10/2021 | Urine | F | 8 | 69 | Auvergne-Rhône-Alpes | Yes - L4 | L. interrogans SG1 |
| 165 | 2021-3678D | Dog | 25/10/2021 | Urine | M | 8 | 70 | Bourgogne-Franche-Comté | Yes - L3 | L. interrogans SG1 |
| 166 | 2021-3886D | Dog | 2/11/2021 | Blood | M | 1 | 64 | Nouvelle-Aquitaine | ND | L. interrogans SG16* |
| 167 | 2021-4247D | Dog | 30/11/2021 | Urine | F | 4 | 71 | Bourgogne-Franche-Comté | Yes - L3 | L. interrogans SG1 |
| 168 | 2021-4358D | Dog | 6/12/2021 | Blood | F | 3 | 1 | Auvergne-Rhône-Alpes | Not | L. interrogans SG1 |
| 169 | 2021-4404D | Dog | 17/12/2021 | Kidney | M | <1 | 29 | Bretagne | ND | L. interrogans SG14* |
| 170 | 2021-4560D | Dog | 22/12/2021 | Blood | M | 1 | 69 | Auvergne-Rhône-Alpes | Yes - L4 | L. interrogans SG16* |
