# Supplementary figures and images for: Genetic diversity of Leptospira strains circulating in humans and dogs in France in 2019-2021
Source: Front Cell Infect Microbiol. 2023 Aug 17;13:1236866. doi: 10.3389/fcimb.2023.1236866 (PMC10469827; doi:10.3389/fcimb.2023.1236866)

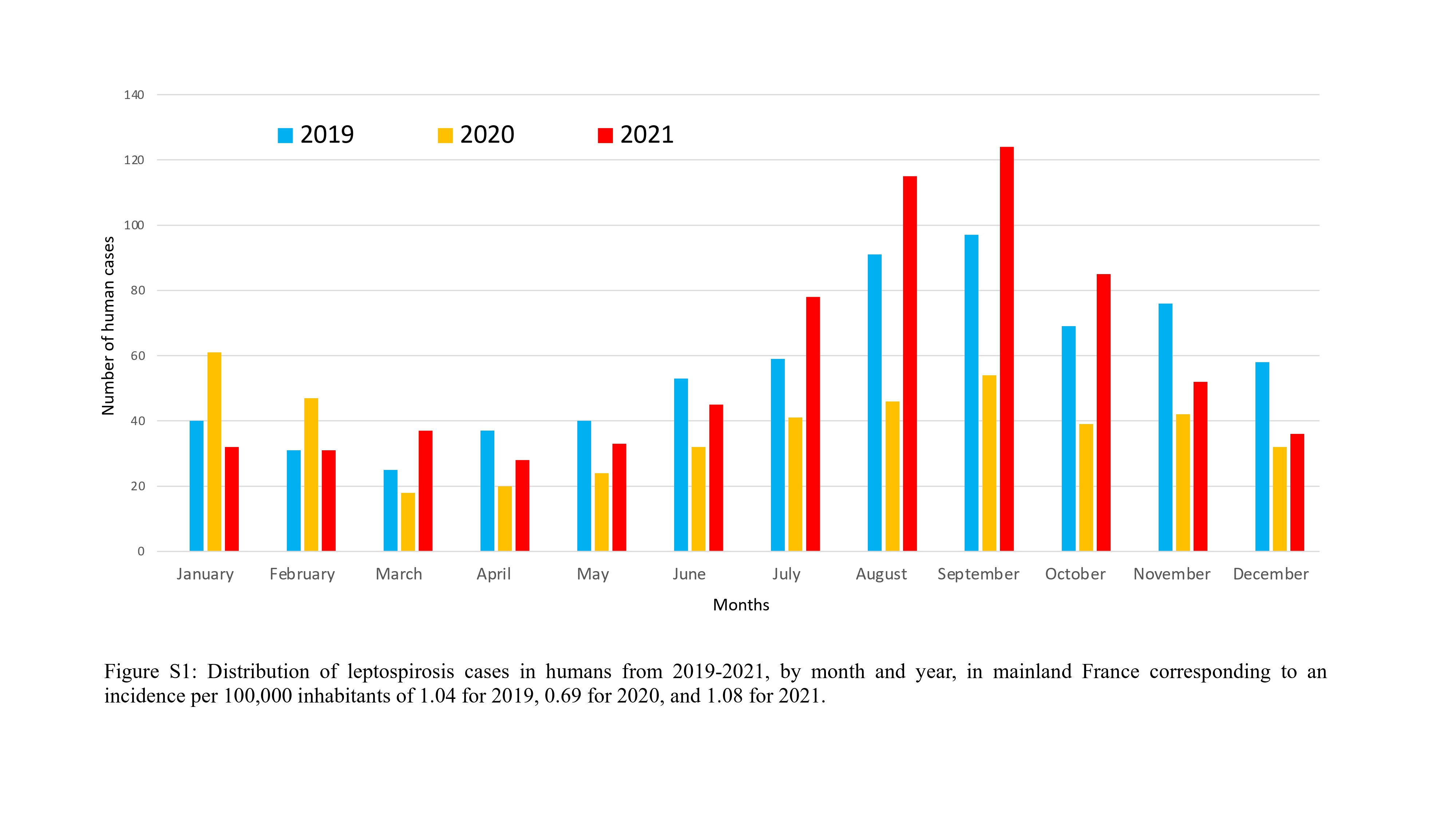

Supplement: Supplementary file 2 [file Image_1.jpg]

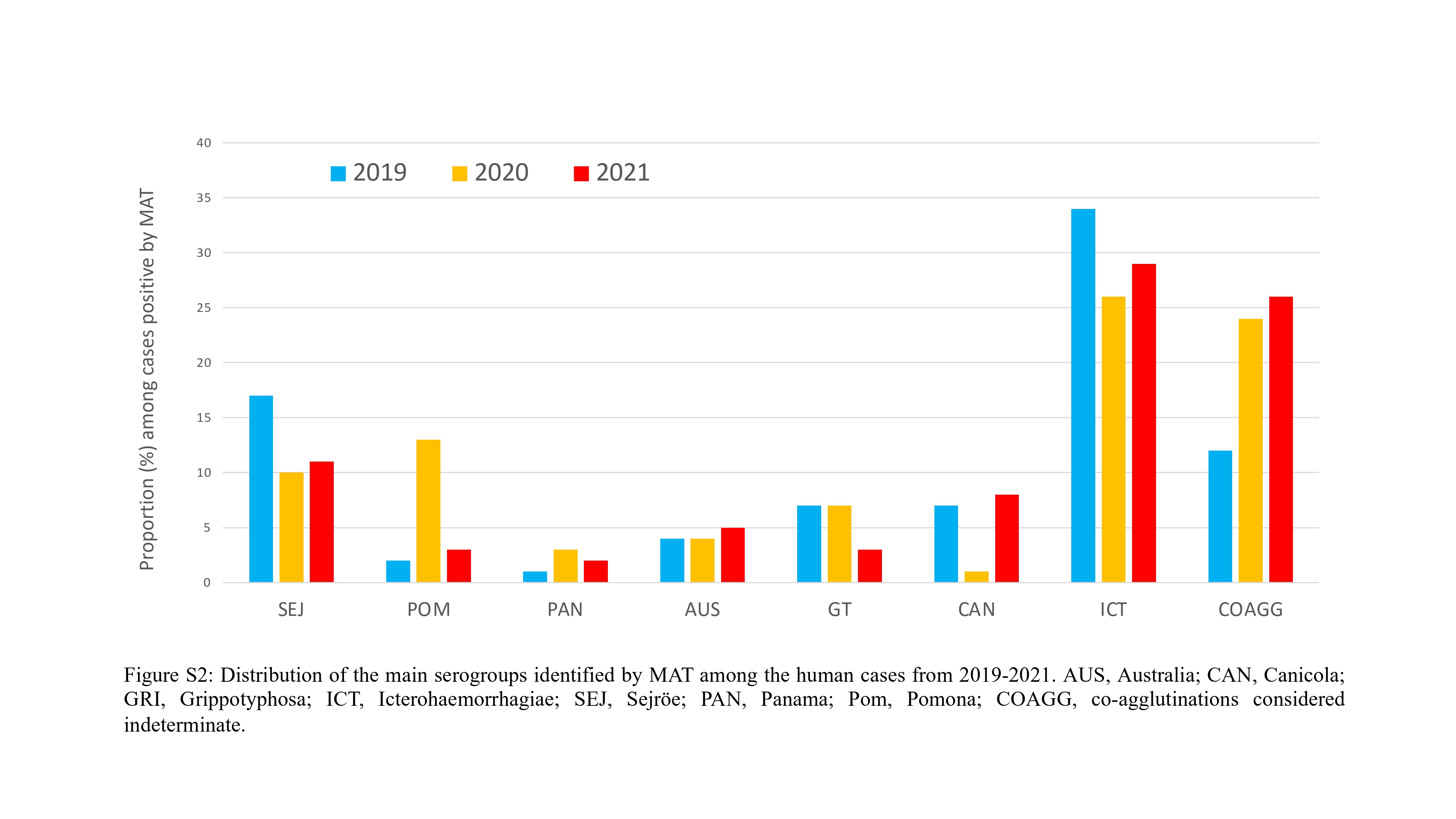

Supplement: Supplementary file 3 [file Image_2.jpg]
